# Sequential Contralateral Large-Vessel Occlusion Following Intravenous Thrombolysis: A Case Report of Dynamic Bilateral Cerebral Arterial Thrombosis

**DOI:** 10.7759/cureus.115675

**Published:** 2026-09-02

**Authors:** M. A. Sayed Joy, Mohsin Fayyaz, Manasi Limaye, Amrita Kiran, Amir A Khan, Ayesha Amjad, Feven A Kenea, Mohammad Siddiqui

**Affiliations:** 1 General Internal Medicine/Neurology/Stroke Medicine, Salford Royal Hospital, Salford, GBR; 2 Medicine, Salford Royal Hospital, Salford, GBR; 3 Respiratory Medicine, Salford Royal Hospital, Salford, GBR; 4 Internal Medicine, Manchester University NHS Foundation Trust, Manchester, GBR; 5 Intensive Care Unit, Salford Royal Hospital, Salford, GBR; 6 Stroke Medicine, Salford Royal Hospital, Salford, GBR; 7 Internal Medicine, Salford Royal Hospital, Salford, GBR

**Keywords:** acute ischemic stroke, carotid artery occlusion, intravenous thrombolysis, large vessel occlusion, mechanical thrombectomy, middle cerebral artery, sequential stroke, thrombus migration

## Abstract

Acute large-vessel occlusion stroke can evolve rapidly during the hyperacute treatment period, occasionally resulting in a clinical and radiological picture that differs substantially from the initial presentation. We report an unusual case of sequential contralateral large-vessel occlusion occurring within approximately three hours. A patient presented with transient dysphasia that resolved rapidly. While awaiting stroke assessment, he developed confusion, left-sided weakness, and dysarthria. Initial imaging demonstrated a distal right middle cerebral artery (MCA) occlusion with a National Institutes of Health Stroke Scale (NIHSS) score of 6. CT angiography (CTA) demonstrated extensive occlusion of the left internal carotid artery (ICA) from its origin through the petrous and intracranial segments; however, the left MCA remained patent. Intravenous thrombolysis was administered, and the patient was transferred for mechanical thrombectomy of the right MCA occlusion. During transfer, he developed sudden complete aphasia and clinical features of a left total anterior circulation syndrome, with deterioration in NIHSS score to 20. Repeat CTA demonstrated recanalisation of the previously occluded right MCA but a new complete occlusion of the previously patent left MCA. The patient underwent emergency left MCA thrombectomy with excellent reperfusion. Neurological recovery was rapid, with an NIHSS score of 2 at 24 hours. The temporal and radiological sequence raised the possibility of distal embolisation or propagation of thrombus from the pre-existing left ICA occlusion following intravenous thrombolysis; however, causality could not be definitively established. Further investigation demonstrated severe left ventricular systolic dysfunction due to non-ischemic dilated cardiomyopathy without definite intraventricular thrombus. Anticoagulation was subsequently commenced. This case is notable for the sequential development of major occlusions in opposite cerebral hemispheres, with documented recanalisation of the initial symptomatic vessel concurrent with emergence of a new dominant-hemisphere occlusion. It highlights the importance of repeated neurological assessment and a low threshold for repeat vascular imaging when the neurological phenotype changes after intravenous thrombolysis. Rapid recognition of a new large-vessel occlusion can permit timely thrombectomy and potentially prevent severe permanent neurological disability.

## Introduction

Acute ischemic stroke due to large-vessel occlusion (LVO) is a time-critical neurological emergency associated with substantial morbidity and mortality. The introduction of endovascular thrombectomy (EVT), in addition to standard medical therapy, has significantly improved functional outcomes in appropriately selected patients with anterior circulation LVO [1,2]. The HERMES (HEart failure Molecular Epidemiology for Therapeutic targetS) individual patient-level meta-analysis demonstrated a substantial reduction in disability with EVT across a broad range of patient characteristics, establishing mechanical thrombectomy as a cornerstone of acute treatment for LVO stroke [3].

Intravenous thrombolysis (IVT) remains an important component of acute reperfusion therapy in eligible patients, including those subsequently undergoing EVT. However, thrombus behavior can change following thrombolytic treatment. Thrombus migration, defined as movement of an occlusive thrombus to a more distal arterial segment, is a recognized phenomenon in patients with acute ischemic stroke undergoing endovascular treatment. In a retrospective analysis of patients undergoing EVT, thrombus migration occurred more frequently among patients who received bridging IVT than among those undergoing direct EVT, suggesting a potential association between thrombolytic therapy and thrombus migration [4].

Conventional thrombus migration typically involves displacement or fragmentation of an existing thrombus to a more distal arterial location. In contrast, the development of a new LVO in the contralateral cerebral circulation represents a distinct and relatively uncommon phenomenon and may reflect a separate embolic event from an arterial or cardiac source [5]. Potential mechanisms include embolization from a pre-existing arterial thrombus or recurrent embolization from a cardiac source. The sequential occurrence of occlusions in opposite cerebral hemispheres, particularly with recanalization of the initial target vessel and emergence of a new contralateral occlusion, presents a diagnostic and therapeutic challenge. Such a scenario may be difficult to recognize if neurological deterioration is attributed solely to progression of the initial infarction or complications of thrombolytic therapy.

We present a case of sequential contralateral middle cerebral artery (MCA) occlusion occurring within approximately three hours. The patient initially presented with a right MCA occlusion in the presence of a concomitant left internal carotid artery (ICA) occlusion, while the left MCA remained patent. Following IVT, the right MCA recanalized, but the patient subsequently developed a new left MCA occlusion associated with a severe dominant-hemisphere syndrome. The case highlights the dynamic nature of thrombus evolution during hyperacute stroke treatment and emphasizes the importance of repeated neurological assessment and prompt repeat vascular imaging when the clinical presentation changes.

## Case presentation

A 71-year-old male patient with a baseline modified Rankin Scale (mRS) score of 1 [6], who was independent in activities of daily living and continued to work and drive, with a past medical history of osteoarthritis and benign prostatism without any significant family history, presented in Accident and Emergency (A&E) with acute neurological symptoms. The patient had an episode of possible amaurosis fugax two days ago. On the day of admission, he woke up with dysphasia, which resolved quickly, and he recovered back to his usual self. His wife found him completely normal and saw him well at 10:30 hours. She then found him confused and slurred with speech at 12:00 hours. 

They arrived in A&E at around 13:15 and were reviewed by the stroke team. He was found to have left-sided mild weakness in the upper limb with inattention to the left with a National Institutes of Health Stroke Scale (NIHSS) score of 6 [7]. Initial non-contrast computed tomography (CT) of the brain showed no acute infarct but showed a hyperdense right MCA. CT angiography (CTA) demonstrated a right MCA M1 occlusion with reduced distal collateral filling. The left ICA was also occluded from its origin; however, the left MCA was normally perfused at this stage (Figure 1 and Figure 2).

**Figure 1:**
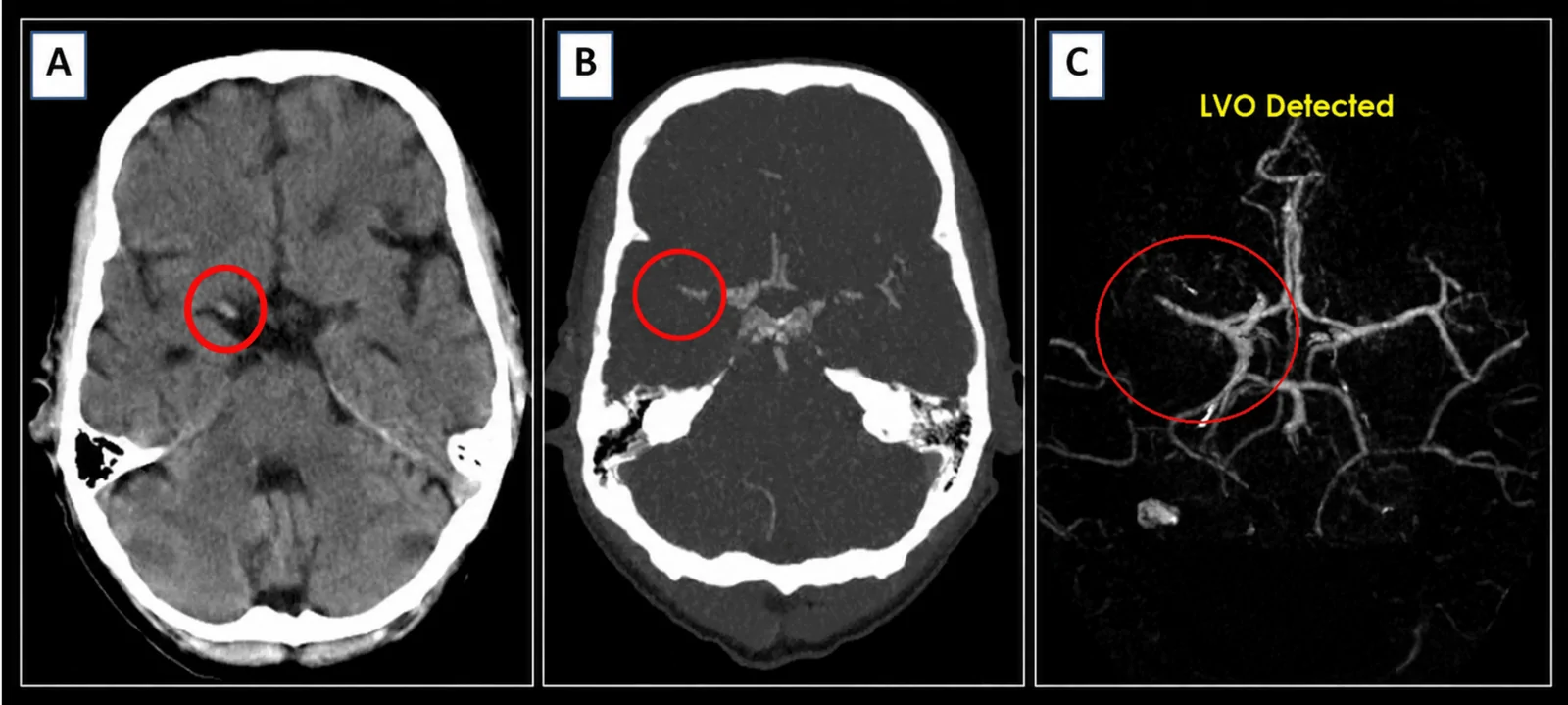
Initial neuroimaging demonstrating the right MCA large-vessel occlusion (A) Non-contrast CT brain demonstrating a hyperdense right MCA (circle); (B) CT angiography demonstrating right MCA occlusion with reduced distal contrast filling (circle); (C) RAPID imaging demonstrating the right MCA large-vessel occlusion (circle) MCA: middle cerebral artery; RAPID: Rapid Processing of Perfusion and Diffusion

**Figure 2:**
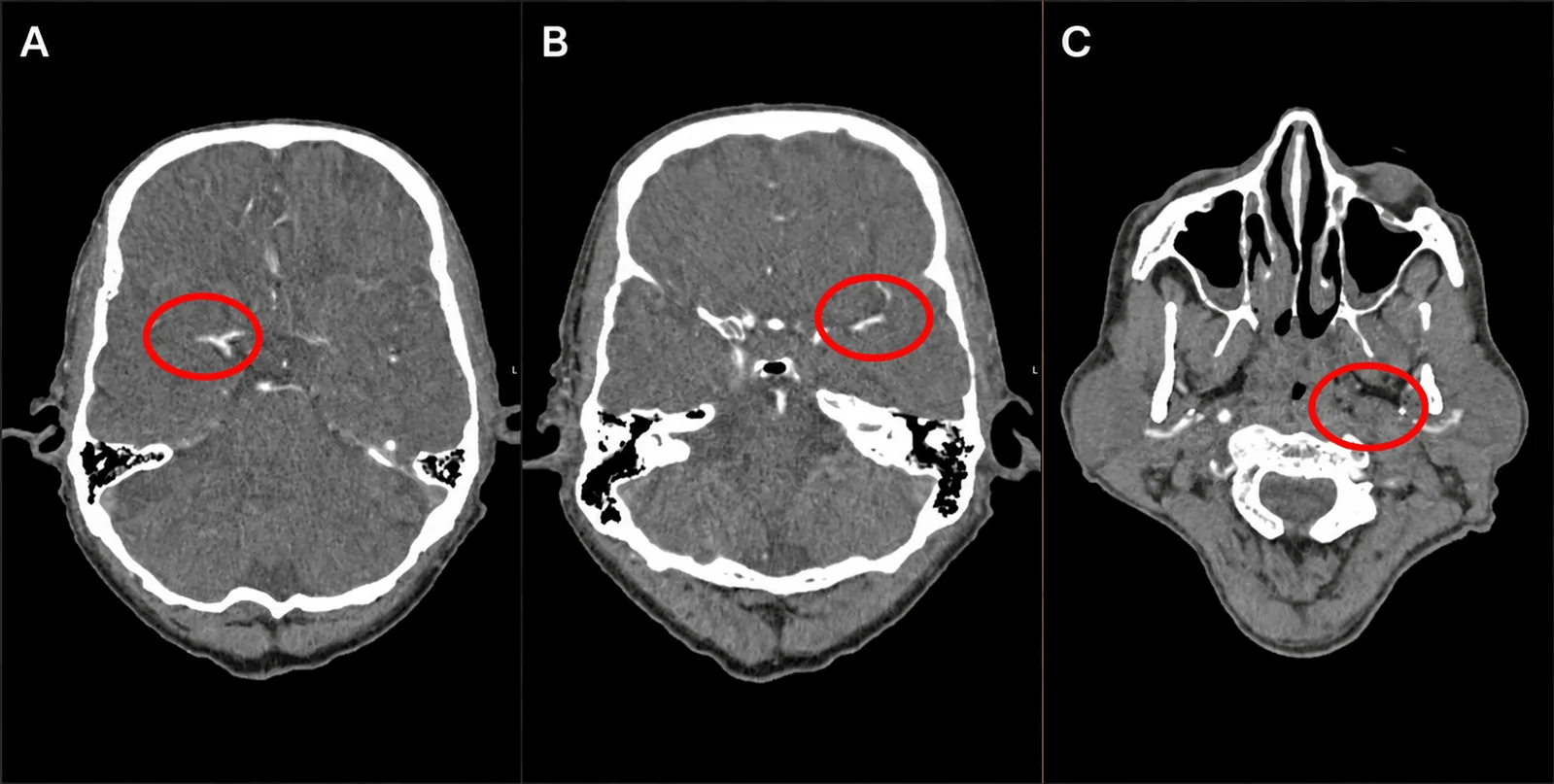
Initial CT angiography from primary stroke centre (A) Right MCA occlusion (circle); (B) Patent left MCA with absent contrast flow in the right MCA (circle); (C) Absent contrast flow in the left ICA, consistent with left ICA occlusion (circle). MCA: middle cerebral artery; ICA: internal carotid artery

Given the right MCA large-vessel occlusion, IVT with tenecteplase was administered at approximately 15:10 hours. The patient was subsequently transferred to a comprehensive stroke centre for endovascular thrombectomy.

During transfer, there was a marked change from the initial right hemispheric syndrome into a severe dominant left hemispheric syndrome. The patient developed a reduction in level of consciousness and new neurological deficits affecting the opposite side. On arrival at the receiving tertiary stroke centre, he had dense right-sided weakness, global aphasia, and hemisensory/hemineglect findings on the right side, with an NIHSS score of greater than 20. The marked change from the initial left-sided syndrome to a severe dominant-hemisphere syndrome raised concern for a new vascular event rather than progression of the initial right MCA stroke.

A repeat CT brain demonstrated a new hyperdense left M1 segment, with preserved left-sided ASPECTS (Alberta Stroke Program Early CT Score) of 10 [8] and evidence of a hyperacute infarct involving the left lentiform nucleus. Repeat CTA demonstrated that the previously occluded right MCA had recanalized, while the left MCA was now occluded involving the M1 and M2 segments (Figure 3 and Figure 4). The previously demonstrated left ICA occlusion persisted but showed partial recanalization, demonstrated by the faint contrast flow (Figure 5).

**Figure 3:**
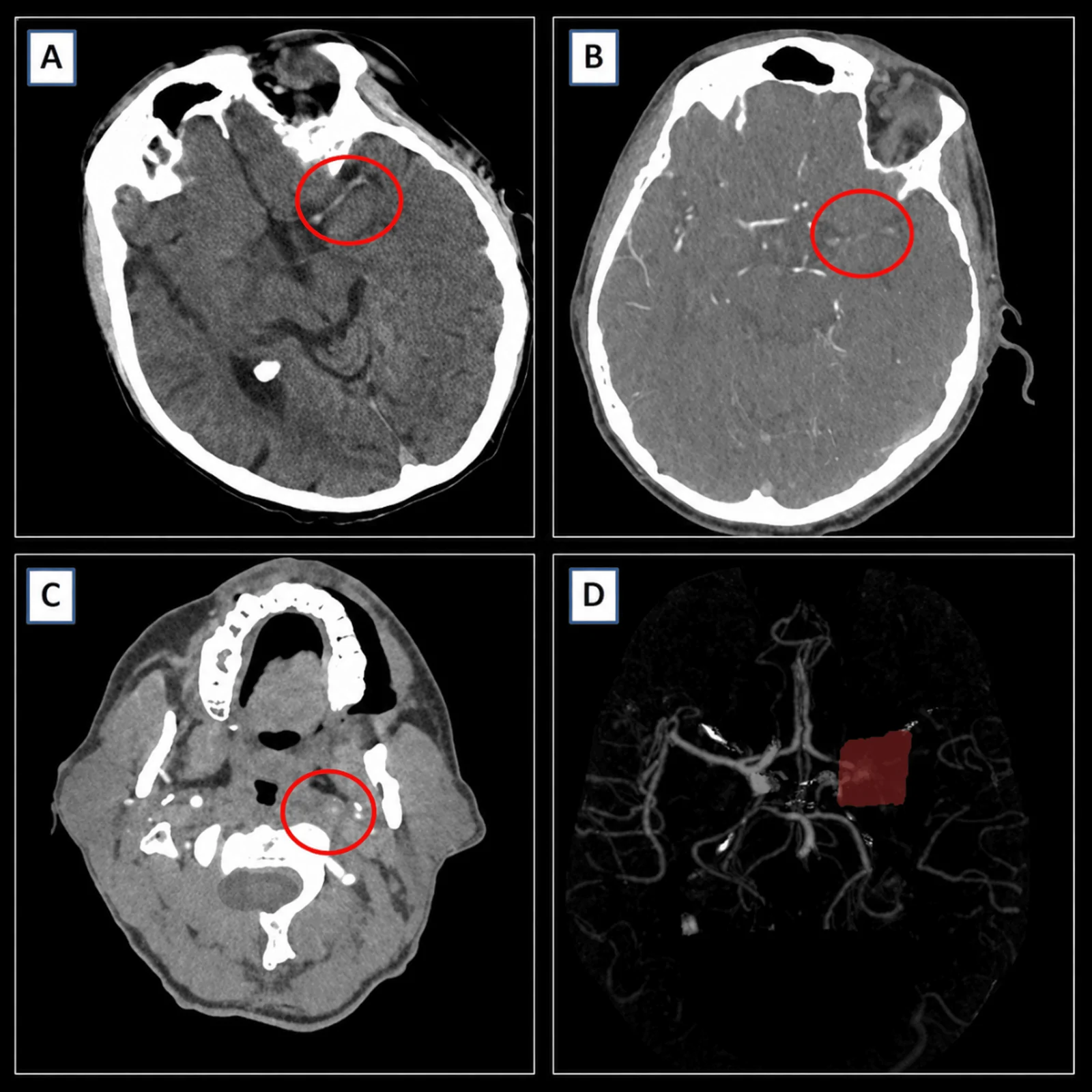
Repeat neuroimaging following acute neurological deterioration demonstrating a new left MCA large-vessel occlusion (A) Non-contrast CT brain demonstrating a new hyperdense left MCA (circle); (B) CT angiography demonstrating absent contrast flow within the left MCA; the circle indicates the site of left M1/M2 occlusion; (C) CT angiography demonstrating persistent occlusion (circle) of the left ICA; (D) RAPID imaging demonstrating the new left MCA large-vessel occlusion. MCA: middle cerebral artery; ICA: internal carotid artery; RAPID: Rapid Processing of Perfusion and Diffusion

**Figure 4:**
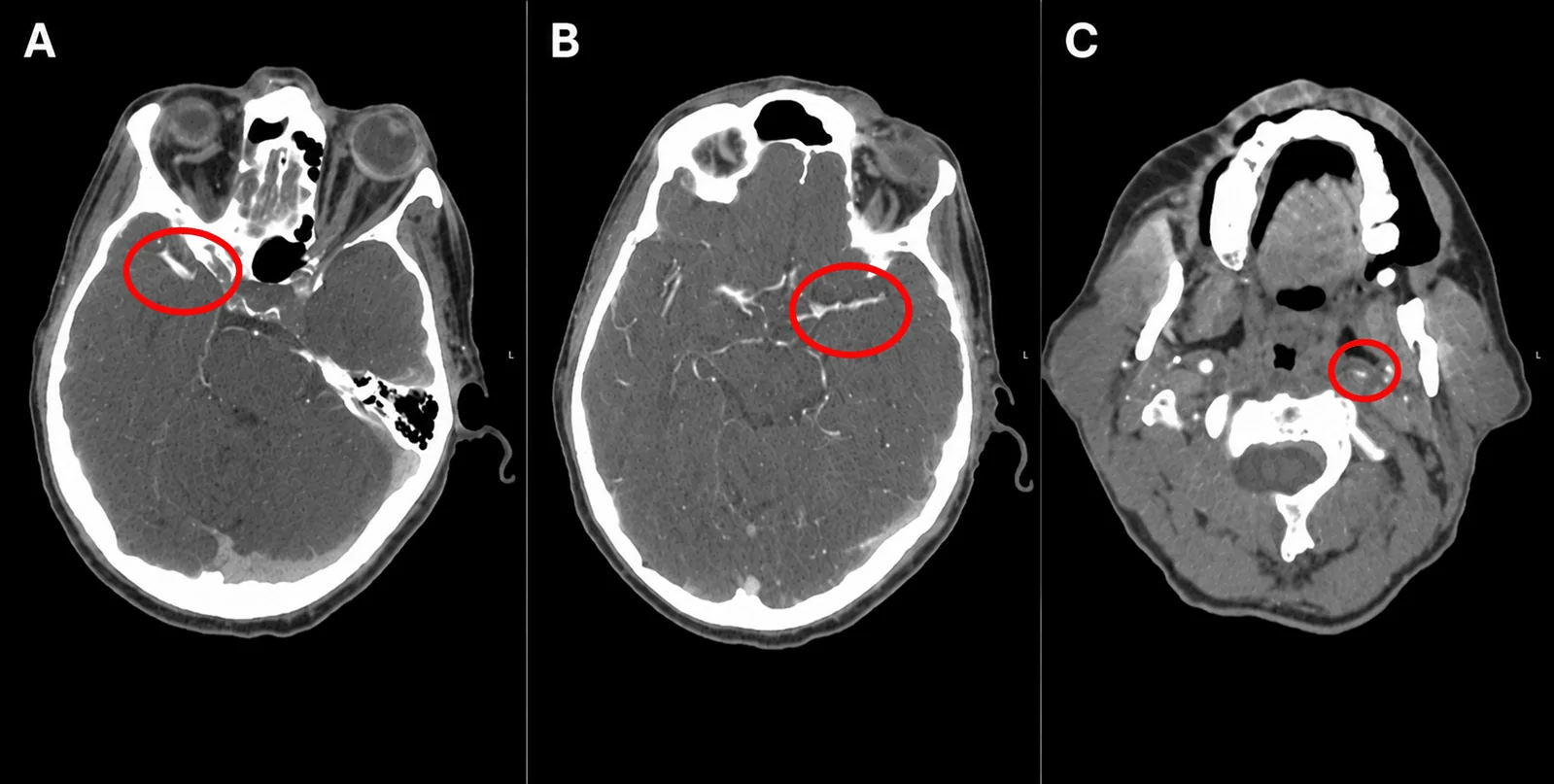
Repeat CT angiography in comprehensive stroke centre following neurological deterioration during transfer demonstrating recanalization of the right MCA and development of a new contralateral left MCA occlusion (A) Restored contrast flow in the right MCA (circle); (B) New left MCA occlusion with preserved distal contrast filling in the right MCA (circle); (C) Persistent left ICA occlusion with faint contrast opacification (circle) MCA: middle cerebral artery; ICA: internal carotid artery

**Figure 5:**
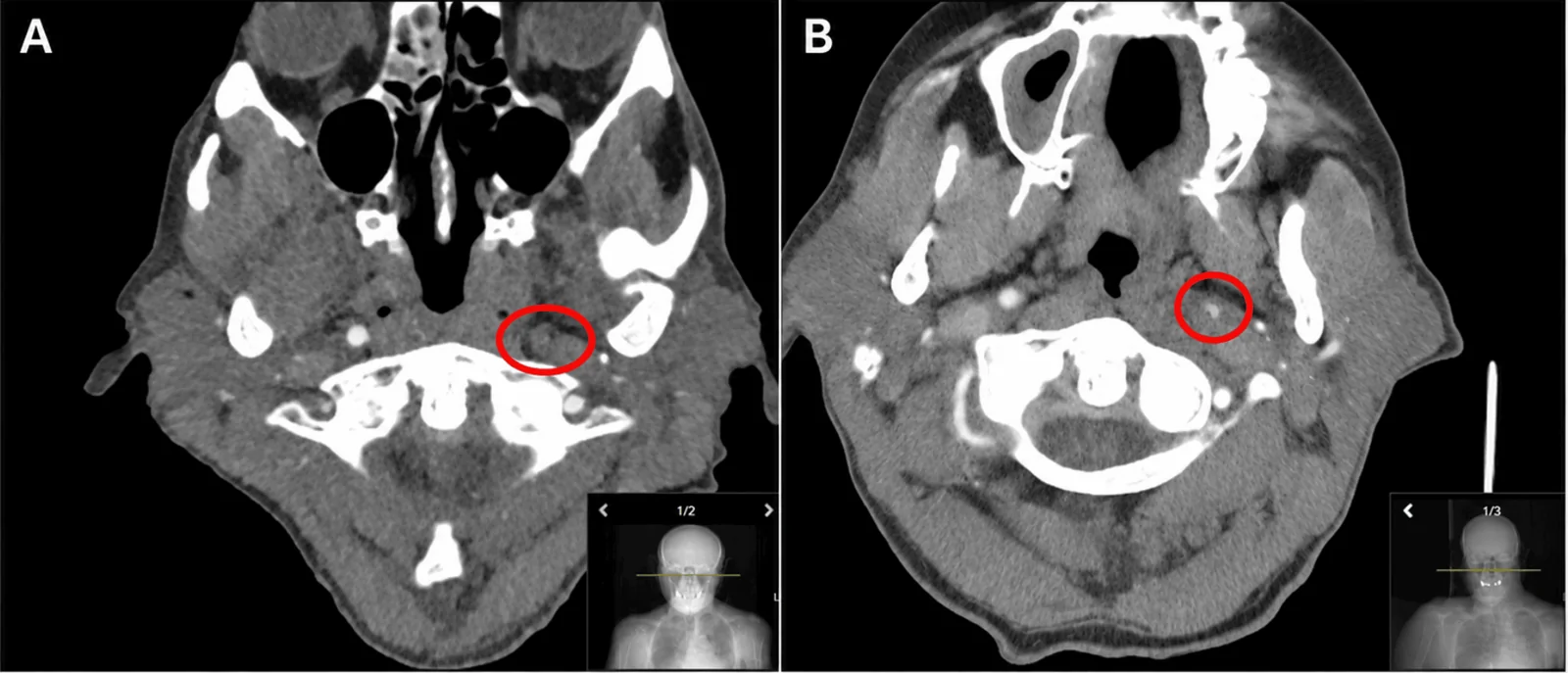
Comparison of the left ICA on CT angiography before and after intravenous thrombolysis (A) Initial CT angiography demonstrating absence of contrast flow in the left ICA (circle); (B) Repeat CT angiography demonstrating faint contrast opacification within the left ICA (circle). This interval change may suggest partial recanalization of the previously occluded vessel; however, its relationship to the subsequent left MCA occlusion cannot be definitively established. ICA: internal carotid artery; MCA: middle cerebral artery

The combination of rapid neurological deterioration, development of a new dominant-hemisphere syndrome, and the marked change in vascular imaging prompted urgent discussion with the interventional neuroradiology team. In view of the new left MCA large-vessel occlusion, the decision was made to proceed with emergency intra-arterial thrombectomy.

Mechanical thrombectomy of the left MCA was performed. Post-intervention angiography demonstrated successful reperfusion (Figure 6) with an angiographic reperfusion grade of 2c (thrombolysis in cerebral infarction (TICI) 2c). [9] As per the interventional radiologist's report, during thrombectomy there was some contrast flow and evidence of soft thrombus in the left ICA, which also showed excellent recanalization following aspiration (Figure 7).

**Figure 6:**
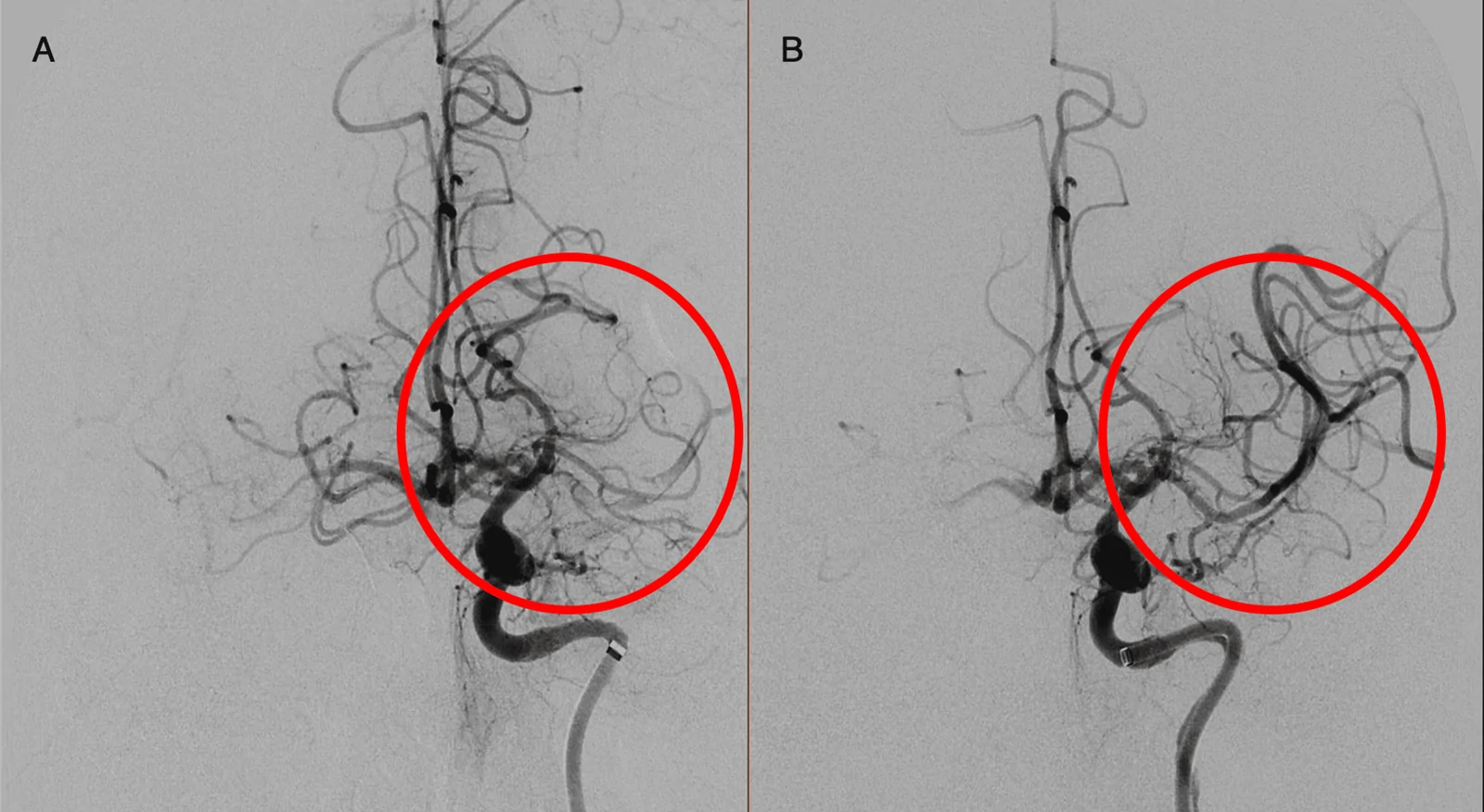
Angiographic images of the left MCA before and after aspiration of clot in mechanical thrombectomy (A) Pre-aspiration angiography demonstrating left MCA occlusion; (B) Angiography demonstrating recanalization of the left MCA following aspiration of the thrombus. MCA: middle cerebral artery

**Figure 7:**
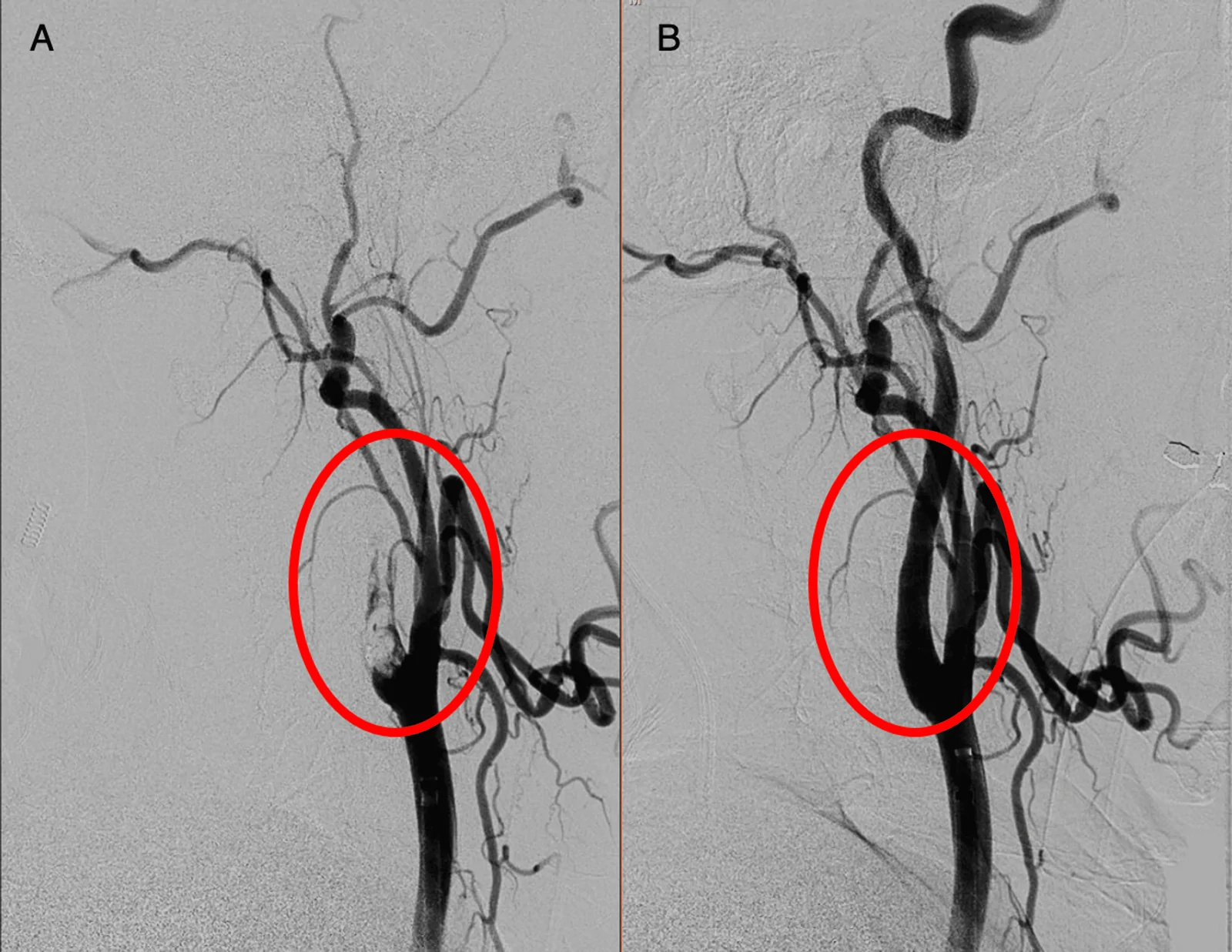
Angiographic images of left ICA during thrombectomy (A) Some contrast flow and soft thrombus; (B) Excellent recanalization of left ICA following aspiration ICA: internal carotid artery

The temporal relationship between the imaging findings raised the possibility that thrombus migration or fragmentation from the pre-existing left ICA occlusion may have contributed to the new left MCA occlusion following thrombolysis. Thrombus migration following IVT has previously been described and may result in a change in the location of arterial occlusion on subsequent vascular imaging [4]. Tenecteplase may also have contributed to thrombus fragmentation or migration in this case. Clot migration following IVT has been reported more frequently with tenecteplase than with alteplase [10]. However, in this patient, a direct causal relationship between tenecteplase administration and the new left MCA occlusion could not be established confidently. Alternative mechanisms, including spontaneous thromboembolism or cardioembolic thrombus progression, were also considered.

The patient demonstrated substantial neurological improvement following thrombectomy. A 24-hour CT brain showed a small acute infarct within the left MCA territory without acute intra- or extra-axial hemorrhage or other hemorrhagic complication. The NIHSS score at 24 hours was 2.

During the admission, further evaluation identified significant cardiac abnormalities. The initial electrocardiogram demonstrated sinus rhythm with ventricular ectopic beats, without sustained atrial arrhythmia. Cardiac biomarkers showed a troponin level of 22.8, while the B-type natriuretic peptide level was markedly elevated at 5,451. Transthoracic echocardiography subsequently demonstrated severe left ventricular systolic dysfunction (LVSD), with a left ventricular (LV) ejection fraction of approximately 18%, severe LV dilatation with global hypokinesia. However, there was no definite LV thrombus identified on the available imaging.

The combination of severe LVSD and multiple arterial occlusions raised concern for a possible cardioembolic mechanism. However, the absence of demonstrable intracardiac thrombus meant that a cardioembolic source could not be confirmed. An urgent heart failure assessment and further investigation of the etiology of the dilated cardiomyopathy were recommended, including consideration of cardiac MRI and contrast-enhanced echocardiography to evaluate for an occult ventricular thrombus.

Initially, antiplatelet therapy was commenced following the acute stroke. Following cardiology and stroke specialist review, anticoagulation with apixaban 5 mg twice daily was subsequently started because of concern regarding a potential cardioembolic source in the setting of severe LVSD. It was noted that, should subsequent cardiac imaging exclude an intracardiac thrombus or other indication for long-term anticoagulation, the secondary stroke prevention strategy would need to be reconsidered.

The patient was discharged with significant neurological recovery and transferred to the local clinical team for continued cardiac investigation, stroke follow-up, and secondary prevention. Subsequently, a cardiac MRI was performed a few weeks later, which was reported as showing "severely dilated LV size and severely impaired systolic function with global hypokinesia. Dilated right ventricle size and severely impaired systolic function. There is bi-atrial dilatation. Left atrial anteroposterior diameter 46 mm". He then went on to have successful implantation of a dual-chamber implantable cardioverter-defibrillator for primary prevention of severe dilated LVSD, non-ischaemic.

## Discussion

The present case illustrates an unusual sequence of rapidly evolving large-vessel occlusions in which the clinical and radiological presentation changed substantially during the hyperacute treatment period. The patient initially had a symptomatic right M1 occlusion, while the left ICA was already occluded but the left MCA remained patent. Following IVT, the right MCA subsequently recanalized, but the patient developed a new left M1/M2 occlusion associated with complete aphasia, dense right-sided weakness, and a marked increase in the NIHSS score from 5 to greater than 20. Emergency thrombectomy of the second occlusion resulted in excellent reperfusion and substantial neurological recovery, with an NIHSS score of 2 after 24 hours.

The most important feature of this case is the sequential and anatomical evolution of the two vascular events. Thrombus migration following IVT is a recognized phenomenon. In a study of patients undergoing endovascular treatment for large-vessel occlusion, thrombus migration was observed in 24.3% of patients and was associated with previous tissue plasminogen activator administration and shorter thrombus length [11]. Similar observations have demonstrated that thrombus location may change between baseline vascular imaging and angiography performed before thrombectomy [12]. Therefore, the concept of thrombus fragmentation or distal migration after thrombolysis is not itself novel.

What makes this case unique is the combination and sequence of events. At the time of the initial presentation, the symptomatic occlusion was in the right MCA despite a concomitant left ICA occlusion. Importantly, the left MCA was still normally perfused. Following thrombolysis, the initial right MCA occlusion disappeared, while the previously patent left MCA became acutely occluded. Thus, the patient did not simply experience distal migration of the original thrombus within the same vascular territory; rather, the dominant clinical syndrome shifted from a right hemispheric presentation to a severe left hemispheric syndrome while the first occlusion simultaneously recanalized. This serial change provides strong temporal evidence that a second large-vessel event occurred during the treatment window.

The presence of a left ICA occlusion does not necessarily imply simultaneous occlusion of the left MCA. Distal cerebral perfusion may be maintained through collateral pathways, including the circle of Willis, allowing the MCA to remain patent despite proximal ICA occlusion. In this patient, preservation of the left MCA on the initial CTA followed by subsequent M1/M2 thrombosis is therefore an important radiological feature. It suggests that the left MCA occlusion was a subsequent event rather than simply a continuation of an already established left MCA occlusion. The initial and repeat vascular studies consequently provide a particularly important element of this case.

The mechanism responsible for the new left MCA occlusion remains uncertain. One possibility is fragmentation or distal embolization of thrombus associated with the pre-existing left ICA occlusion following thrombolysis. The temporal relationship is compatible with this explanation: the right MCA recanalized following thrombolysis, while a new left MCA occlusion developed shortly thereafter. However, causality cannot be established from temporal association alone. Spontaneous embolization from the left ICA, progression of the underlying thrombotic process, or an independent embolic event are alternative explanations. The case should therefore be interpreted as demonstrating a strong temporal association between thrombolytic treatment, recanalization of the initial occlusion, and emergence of the contralateral occlusion rather than definitive proof that thrombolysis caused the second occlusion.

Another important consideration is the discovery of severe non-ischemic dilated cardiomyopathy. Transthoracic echocardiography demonstrated a severely impaired LV ejection fraction of approximately 18%, severe LV dilatation and global hypokinesia, biatrial enlargement, impaired right ventricular function, and moderate mitral regurgitation. Severe LV systolic dysfunction provides a plausible substrate for thromboembolism, particularly if an intracardiac thrombus is present. Previous work in patients with dilated cardiomyopathy has demonstrated an increased risk of stroke among patients with echocardiographically identified LV thrombus [13]. Nevertheless, no definite LV thrombus was identified in this patient, and the absence of a demonstrated thrombus means that a cardioembolic source cannot be confirmed.

The possibility of an occult cardiac thrombus remained clinically relevant because routine transthoracic echocardiography may not reliably exclude LV thrombus, particularly when image quality is limited or thrombus morphology is unfavorable. Cardiac MRI and contrast-enhanced echocardiography can provide additional sensitivity for LV thrombus detection in selected patients [14,15]. Accordingly, further cardiac investigation was recommended in this case, including consideration of contrast-enhanced echocardiography and cardiac MRI. This is particularly important because identification of a cardioembolic source would influence both secondary stroke prevention and the interpretation of the bilateral arterial events.

The management of the second neurological deterioration is another important learning point. The patient's neurological syndrome changed dramatically during transfer, from left-sided weakness and dysarthria to complete aphasia, dense right-sided weakness, and neglect. This change in phenotype was a critical clue that the initial stroke syndrome no longer explained the patient's clinical state. Repeat CT and CTA demonstrated not only the new left MCA occlusion but also recanalization of the previously occluded right MCA. Without repeat vascular imaging, the second large-vessel occlusion could potentially have been missed or attributed to progression of the initial stroke.

This case therefore emphasizes that neurological deterioration after IVT should not automatically be attributed to infarct progression or treatment-related complications. When the neurological phenotype changes, particularly when new deficits localize to the contralateral hemisphere, repeat vascular imaging should be considered urgently. In this patient, recognition of the new dominant-hemisphere LVO allowed immediate thrombectomy and was followed by near-complete neurological recovery.

The case also demonstrates the potential value of serial vascular imaging in understanding dynamic thrombus behavior. A single CTA provides only a snapshot of the cerebrovascular state. In patients with multiple simultaneous arterial abnormalities, comparison of sequential vascular studies may reveal an evolving thromboembolic process that would otherwise be difficult to recognize. In the present case, the transition from right MCA occlusion to right MCA recanalization with new left MCA occlusion provides the central radiological evidence supporting the occurrence of sequential rather than simultaneous symptomatic LVOs.

There are several limitations to the proposed mechanism. First, the left ICA occlusion was present on the initial CTA, but a direct demonstration of thrombus fragmentation from the left ICA into the left MCA was not possible. Second, severe LV systolic dysfunction provided an alternative potential embolic source, although no definite intracardiac thrombus was detected. Third, the temporal association with tenecteplase does not prove causality because spontaneous thrombus migration or embolization could have occurred independently. Finally, this is a single case and therefore cannot establish the frequency or probability of this phenomenon.

This case illustrates the diagnostic value of correlating serial neurological examinations with sequential vascular imaging in patients whose clinical presentation changes during hyperacute stroke treatment.

## Conclusions

This case demonstrates the dynamic evolution of large-vessel occlusion during hyperacute stroke treatment, with recanalization of the initial right MCA occlusion followed by the development of a new contralateral left MCA occlusion. Although the second occlusion occurred after IVT, this temporal association does not establish causality, and several mechanisms remain plausible, including embolization from the pre-existing left ICA lesion, recurrent cardioembolism, or an ongoing thromboembolic process. The case highlights that an unexpected change in neurological phenotype should prompt urgent repeat vascular imaging to identify a new treatable occlusion or bleeding. Rapid recognition and thrombectomy of the second LVO resulted in excellent neurological recovery.
